# Strategies for the source protection of data assets on processing techniques for intelligent manufacturing in traditional Chinese medicine

**DOI:** 10.3389/fpubh.2026.1832910

**Published:** 2026-07-23

**Authors:** Siyue Chen, Jinlian Zhang

**Affiliations:** 1 Zhongnan University of Economics and Law Center for Studies of Intellectual Property Rights, Wuhan, China; 2 Jiangxi University of Traditional Chinese Medicine, Nanchang, China

**Keywords:** benefit-sharing, data elements, digital watermarking, intellectual property, TCM processing

## Abstract

The digital transformation of traditional Chinese medicine (TCM) processing techniques currently faces a critical industry bottleneck: the lack of rights protection for source knowledge contributors, which results in an insufficient supply of high-quality data. Applying principles from information economics and algorithmic theory, this study analyzes risk mechanisms where physical control fails during technical externalization and institutional safeguards are undermined by algorithmic “black box” complexity. To address these vulnerabilities, a full-cycle safeguarding system was established through a synergy of institutional and technological measures. This analysis suggests that institutional data intellectual property registration may clarify ownership and facilitate benefit-sharing, while technological implementations - specifically transformation-domain digital watermarking and model backdoor detection - offer a promising pathway toward robust data traceability. This integrated strategy has the potential to address the challenges of safeguarding contributor rights and could activate the endogenous motivation for data supply. Consequently, this framework may provide a foundation for fostering new productive forces and supporting the sustainable, high-quality development of the intelligent TCM manufacturing industry.

## 1 Introduction

The modernization of traditional Chinese medicine has entered a period of digital and intelligent transformation. In July 2024, the National Administration of Traditional Chinese Medicine and the National Data Bureau jointly issued the “Several Opinions on Promoting the Development of Digital Traditional Chinese Medicine,” explicitly stating the need to make every effort to build “Digital and Intelligent Traditional Chinese Medicine” and planning to use 3–5 years to promote the deep integration of emerging technologies such as big data and artificial intelligence into the entire chain of inheritance and innovation in traditional Chinese medicine (1). In the field of Chinese herbal medicine processing, utilizing digital methods to encode traditional processing techniques and driving the digital and intelligent transformation of herbal medicine processing is a key lever for implementing the Digital China strategy and achieving high-quality development in TCM.

As a national-level intangible cultural heritage, the core of traditional Chinese herbal preparation lies in the precise control of “heat control” by experienced herbalists. This craft essentially falls under what Polanyi termed “tacit knowledge,” characterized by a high degree of individual dependency and non-standardization (2). Consequently, traditional Chinese herbal preparation has long faced the dilemma of “the craft dying with the master” in terms of succession. Algorithmic technology provides support for making this “tacit knowledge” explicit. Specifically, sensors can be used to parameterise the veteran herbalists’ subjective experience regarding “heat control,” thereby encoding it as knowledge data. After deep learning processing, this is fixed as a model and, combined with external mechanical devices, enables algorithm-driven automated processing (3). Compared to traditional TCM processing techniques, algorithm-based methods offer greater physical and chemical uniformity and higher reproducibility, representing a crucial pathway for the transformation of TCM processed products from manual production to industrial-scale manufacturing. Furthermore, the digital preservation of veteran practitioners’ experience ensures that this knowledge gradually becomes public domain during the dissemination of technology, thereby eliminating the phenomenon of “skills dying with the master.”

With technological advancements, the integration of artificial intelligence with TCM has deepened progressively, with application scenarios permeating an extensive range of domains, including intelligent identification of medicinal materials, screening of bioactive constituents, decoding of formulaic compatibility patterns, and quality control in pharmaceutical manufacturing. Within this landscape, the intelligent stir-frying machine serves as a quintessential hardware embodiment of this convergence (4). However, the digital and intelligent transformation of the TCM processing industry still faces severe challenges. At present, the core contradiction within the industry is no longer a lack of hardware equipment or technological accumulation, but rather a scarcity of high-quality data resources. The fundamental cause lies in the fact that the rights and interests of the original contributors - the veteran herbalists - have not been adequately safeguarded. In the process of transforming data into assets, the rights of veteran herbalists comprise both intellectual and economic interests. Intellectual interests primarily manifest as the source contributors’ rights to attribution and informed consent, while economic interests primarily manifest as the right to control the data and share in the profits following the inclusion of the data assets in financial statements and the commercial application of models.

Where the mechanisms safeguarding these rights fail, traditional pharmaceutical workers face the risk of “de-sourcing” during the digitization of “tacit knowledge.” This includes being replaced internally after providing knowledge data, as well as suffering loss of benefits due to external replication and infringement resulting from the non-exclusive nature of data. Against this backdrop, due to a lack of security and a sense of fulfillment, veteran herbalists - operating under the assumption of “economic rationality” - tend to adopt “defensive supply strategies” during data collection. These include outright refusal to provide data, passive cooperation, or withholding core skills while wearing multimodal sensors as a form of resistance. This logically results in a discrepancy between the time-series temperature data collected by enterprises and the actual optimal processing curve, potentially leading to insufficient robustness in the trained algorithmic models. In such scenarios, early-stage intelligent devices may exhibit a finished product pass rate far lower than that of manual processing when handling complex herbal materials, and their model generalization capabilities could be severely lacking, posing a potential constraint on the industrial application of algorithmic TCM processing methods.

Having identified the internal and external risks associated with the involvement of experienced herbalists in the R&D of algorithmic TCM processing methods, we must further elucidate the underlying mechanisms of these risks. Embodied experience is naturally protected due to its strong dependence on the individual, but once encoded and parameterized, it becomes knowledge data, possessing the characteristics of a general data production factor. However, due to the opacity of algorithmic black boxes obscuring the R&D process, existing behavioral control mechanisms within the institutional framework have failed. Consequently, this paper argues that efforts should begin with the registration of data intellectual property rights, aiming to achieve governance at the source through the establishment of ownership. Subsequently, relying on “digital watermarks” as a technical safeguard, full traceability of knowledge data throughout its lifecycle may be realized. The ultimate goal is to activate internal industrial incentive mechanisms for the supply of source data, potentially providing sustainable momentum for the digital and intelligent upgrading of the traditional Chinese medicine processing industry.

## 2 Risk characteristics of the digital and intelligent transformation of traditional Chinese medicine processing techniques

### 2.1 Technical context: taking the algorithmic transformation of the Bai Jiang Dan processing technique as an example

The core step in the digital and intelligent transformation of traditional Chinese medicinal processing techniques involves parameterizing the subjective experiential knowledge of veteran practitioners regarding these techniques and utilizing deep learning to identify the objective relationships between these parameters. Taking the Bai Jiang Dan preparation process as an example, it primarily employs the “ascending method” to refine mercury, potassium nitrate, white alum, green alum and salt into block-shaped crystals (5), involving stages such as material preparation, initial heating, sealing, mercury reduction, mercury collection and removal of fire toxicity (6). The most critical aspect is the control of heat intensity, namely the use of low heat to activate the materials, high heat to catalyze the reaction, and final heat to ensure complete reaction of the mercury (7). Yang Yu et al. (8) optimized the Bai Jiang Dan preparation process, noting that the traditional method lacked uniform standards across its various stages and that the use of charcoal and sand trays made it difficult to control the heat. They employed the Analytic Hierarchy Process (AHP) combined with the entropy weighting method to quantitatively optimize the refining parameters, ultimately determining the optimal preparation parameters (8). With the assistance of experienced herbalists well-versed in Bai Jiang Dan processing, the application of algorithmic methods to digitize and intelligentise the Bai Jiang Dan preparation process can facilitate a paradigm shift in research. In the aforementioned studies, an infrared thermal imaging platform was primarily used to record changes in the surface temperature of the processing vessel, while multiple methods were employed to quantitatively analyze changes in material elements, phase transformations and thermal properties before and after processing. Using the same technical approach, it is possible to acquire thermodynamic matrix data throughout the entire process during preparation by experienced herbalists, thereby generating two datasets: one for temperature variation parameters and the other for preparation outcome parameters. Through training using convolutional neural networks or long short-term memory networks, the algorithm can extract “temperature control-crystal structure” correlation features from the thermal imaging data, thereby fitting a dynamic temperature control curve that yields the optimal preparation outcome. When combined with an external temperature control device, this forms an intelligent processing machine centred on an algorithmic Bai Jiang Dan preparation method. By inputting raw material property data and real-time thermal imaging data, and outputting the applicable temperature curve, it achieves precise control of the cooking process under algorithmic control. This process is no longer based on experimental pharmaceutical research, but rather directly encodes the lifetime of Bai Jiang Dan processing experience accumulated by veteran pharmacists, transforming embodied “tacit knowledge” into disembodied knowledge data.

### 2.2 Internal risks: the conflict between one-off knowledge data provision and sustained knowledge returns

Throughout the process of digitizing and intelligentizing traditional Chinese medicine processing techniques, veteran herbalists - as the custodians of these traditional skills - serve as the primary source of high-quality training data. On the one hand, the datasets used for algorithm training are derived from the collection of parameters recorded during the operations of these veteran herbalists. During the preparation of Bai Jiang Dan, veteran herbalists, drawing on their experience in controlling heat, switch between gentle and vigorous heating in a timely manner, thereby imparting key temporal characteristics to the thermodynamic matrix data. This enables the algorithm to uncover the correlation between “temperature control and crystal structure.” On the other hand, the algorithm’s reinforcement learning based on human feedback also requires the judgment of veteran herbalists. During the intelligent processing phase, experienced herbalists must evaluate the trial runs of intelligent equipment, helping to construct a reward model by labeling outcomes as “good” or “bad,” thereby calibrating algorithmic training and decision-making biases. It can be argued that veteran herbalists, as the source of knowledge, are a prerequisite for the digital and intelligent transformation of traditional Chinese medicine processing techniques. However, the provision of knowledge data by veteran herbalists is a one-off act; once algorithm training is complete, pharmaceutical companies fully acquire the encoded results of the veteran herbalists’ “tacit knowledge,” at which point the irreplaceability of the veteran herbalists diminishes significantly (9). In subsequent algorithm iterations, as the number of model parameters grows exponentially, the marginal contribution of the original experiential data decreases. Under the current industrial model, the data contributions of veteran herbalists are often treated as part of their “duties” or acquired through one-off contractual buyouts, thereby depriving them of the right to share in the profits derived from the resulting data assets. This logically creates a misalignment between value creation and benefit distribution: while algorithmic formulation methods continue to generate value, the veteran formulators - as the source of that knowledge - face a structural risk of being marginalized and facing internal “de-sourcing.” This can be understood as the underlying motivation for veteran formulators to adopt “defensive supply strategies” and the core reason for the suppression of the endogenous drive to supply high-quality data.

### 2.3 External risks: the conflict between high R&D costs and the ease of unfair competition

The digital-intelligent transformation of Traditional Chinese Medicine (TCM) processing techniques constitutes a highly complex systemic undertaking. Given the pronounced embodied character of traditional processing expertise - that is, tacit knowledge inextricably embedded in the practitioner’s corporeal experience and perceptual judgment - the conversion of such expertise into multimodal datasets confronts formidable technical barriers. Compounding this challenge is the inherently nonlinear nature of the transformation process, which necessitates the deployment of computationally intensive deep learning models and the assembly of interdisciplinary teams (10). Consequently, the attendant upfront sunk costs are substantial, rendering this a paradigmatic instance of resource-intensive engineering. In contrast, the costs of replicating and transferring core data elements are minimal. Competitors can obtain cleaned and annotated empirical datasets through improper means and train models with identical performance with virtually zero R&D investment, creating a significant “free-riding” effect across the entire industry. Although algorithmic TCM processing methods, as a technical solution, are eligible for patent protection provided they meet the three requirements of patentability, under the current institutional framework, the scope of patent rights extends only to the algorithmic architecture itself and cannot readily cover the source training data that forms the core of the model (11). Competitors can utilize the data to train neural networks with different architectures, thereby circumventing patent infringement, while veteran herbalists are unable to assert their rights. Moreover, compared to research and development, stealing knowledge and data through unfair competition is clearly a lower-cost option. This protection gap can prevent veteran herbalists from controlling the external circulation of their data, thereby potentially depriving them of the appropriate financial returns from third-party commercial exploitation, resulting in a further misalignment between value creation and profit distribution.

## 3 Analysis of the risk mechanisms in the digital and intelligent transformation of traditional Chinese medicine processing techniques

### 3.1 Failure of physical control: separation of ownership and possession of data assets

**Figure 1:**
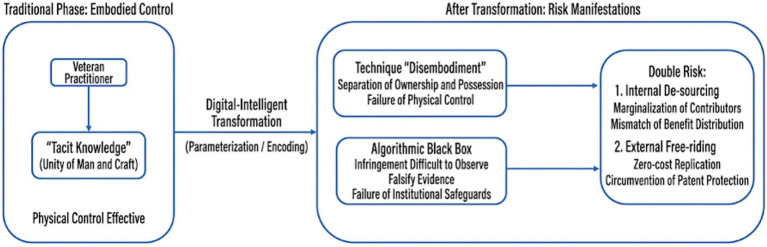
Risk mechanisms of the digital and intelligent transformation of TCM processing techniques.

Traditional Chinese herbal preparation techniques, as “tacit knowledge” possessed by veteran herbalists, exhibit distinct “embodied” characteristics. These techniques stem from the subjective experience accumulated through the long-term practice of veteran herbalists and are inseparable from their physical persons. This physical nature enables veteran herbalists, through control over their bodies, to naturally possess exclusive rights to the techniques, exercising absolute control over the knowledge assets, with knowledge transmission achievable only through the personal instruction and demonstration of the veteran herbalists themselves. Following the digital and intelligent transformation of these processing techniques, the process of algorithmization is essentially one of “disembodiment.” The experience of veteran herbalists is encoded into machine-readable parameters, transforming the nature of the asset from subjective experience into a data production factor, characterized by “non-rivalry” and “non-exclusivity” (12). According to information economics theory, the use of data elements does not cause any depletion of the data itself; attempting to prevent others from using the data through possession is extremely costly and difficult (13). In other words, once data is generated, the cost of replication approaches zero. This leads to a separation of ownership and possession of knowledge data; traditional pharmacists lose the ability to control the dissemination of their skills through physical possession of knowledge, and are unable to exercise effective control over data assets directly. Consequently, they cannot safeguard their own interests by blocking the external circulation of data; the specific mechanism of risk evolution is illustrated in Figure 1.

### 3.2 Failure of institutional protection: infringement concealed by the “algorithmic black box”

Within the existing institutional framework, the rules providing rights protection for traditional herbalists primarily consist of internal contractual provisions and external trade secret protection rules, both of which presuppose that traditional herbalists possess the capacity to observe and provide evidence of infringing acts. However, due to the unique nature of digital and intelligent technologies, the digital and intelligent transformation of traditional Chinese medicine processing techniques is obscured by the “algorithmic black box,” creating a cognitive barrier (14). As “tacit knowledge,” the preparation of traditional Chinese medicine inherently involves a certain degree of cognitive opacity. Testing the prepared products cannot reveal the specific parameters used during the preparation process. The digital and intelligent transformation of traditional Chinese medicine preparation techniques has not opened this cognitive black box; rather, it has further compounded it with an “algorithmic black box.” Deep neural networks primarily utilize a vast number of weight parameters and bias terms to store and express knowledge within a high-dimensional vector space, exhibiting distributed and non-linear characteristics. Although these parameters are readable by machines, this form of knowledge representation is highly opaque to humans, making it impossible to establish an explicit mapping relationship between parameter values and specific processing outcomes based on logic (15). The superimposition of these two “black boxes” further obstructs observation and evidence-gathering. In the digital and intelligent manufacturing industry, physical and chemical rules are transparent “white boxes.” Following an infringement, even if the process is obscured by the “algorithmic black box,” preliminary proof can still be established by reverse-engineering the results and comparing the machining logic, such as the cutting paths of machine tools. In the context of digitalized and intelligent traditional Chinese medicine preparation, experienced herbalists are virtually unable to penetrate the black box during the process to verify whether the training dataset contains their own knowledge-based data, nor can they reverse-engineer the preparation parameters used in model training by analyzing the final processed product. Technology effectively creates information barriers, which may prevent veteran herbalists from overseeing data usage. Given the inability to observe the inner workings of the “algorithmic black box,” veteran herbalists face significant obstacles in proving the existence of a breach of contract or providing preliminary evidence of trade secret theft, which can render the aforementioned rules for safeguarding rights ineffective (16).

## 4 Full-cycle governance of knowledge data in the digital and intelligent transformation of traditional Chinese medicine processing techniques

### 4.1 Source governance: establishing rights through intellectual property registration of knowledge data

**Figure 2:**
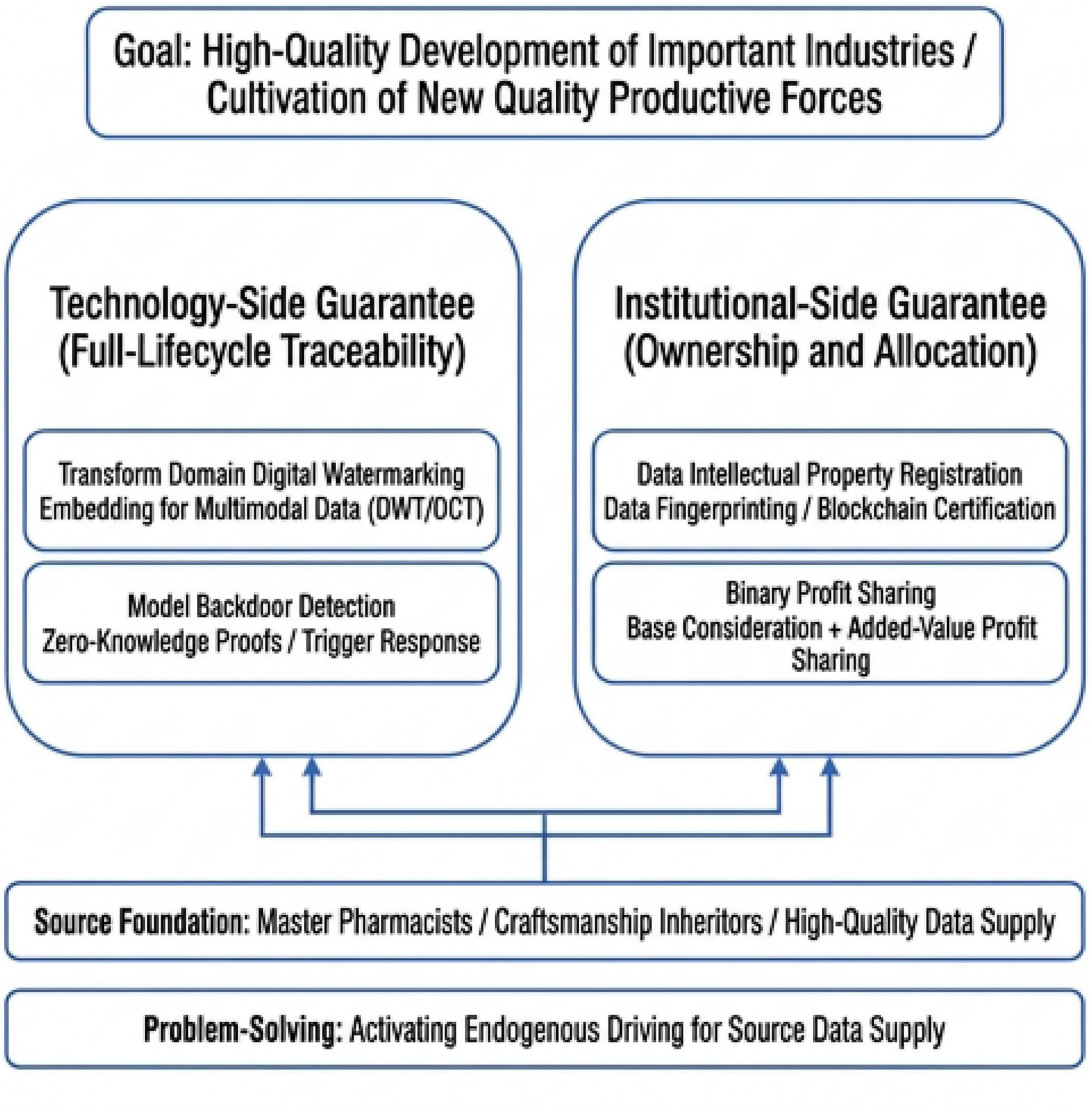
A “technology-institutional” collaborative life-cycle protection framework for TCM intelligent manufacturing.

As shown in Figure 2, this paper constructs an integrated “technology-institution” collaborative safeguard system. Source governance serves as the logical starting point. It should thoroughly implement the requirements of the “Twenty Provisions on Data” regarding the establishment of a data property rights system, and fully utilize the pilot achievements of “data intellectual property registration” promoted by the National Intellectual Property Administration in Zhejiang, Beijing and other regions to construct a data rights confirmation paradigm tailored to the characteristics of the traditional Chinese medicine industry (17). According to the latest data released by the China National Intellectual Property Administration in January 2026, as of October 2025, pilot regions across the country had collectively issued over 40,000 data intellectual property registration certificates, with financing guarantees and licensing transactions amounting to nearly 15 billion yuan. Data intellectual property registration has thus progressed from local pilot schemes to large-scale application, becoming a mature pathway for safeguarding the rights and interests of data elements (18).

Within the WIPO framework, strategies for the protection of traditional knowledge are conventionally bifurcated into two categories. The first is defensive protection, which is designed to preclude external actors from erroneously obtaining patent rights over traditional knowledge - a paradigmatic example being India’s Traditional Knowledge Digital Library (TKDL), which functions as a prior art reference base in patent examination proceedings. The second is positive protection, exemplified by the grant of rights to traditional knowledge holders through sui generis specialized regimes (19). With respect to veteran TCM practitioners, the protection of their individual experiential datasets through data intellectual property registration constitutes, in essence, a form of positive protection. At the level of concrete legal instruments, the EU Data Act establishes the foundational rule that “data generators are entitled to data rights,” by conferring upon users of connected products the rights of access to and sharing of data generated by their devices (20). Although the primary application context of the EU Data Act is industrial Internet of Things environments, and it does not directly address traditional knowledge, the underlying normative logic shared by both regulatory domains remains consistent: both recognize that data generators and contributors may assert rights grounded in their cognitive investment and labor inputs, with protection to be effectuated through empowerment mechanisms. Furthermore, the WIPO Treaty on Intellectual Property, Genetic Resources and Associated Traditional Knowledge (GRATK Treaty) introduces a mandatory disclosure of origin obligation in patent applications, requiring the establishment of source identifiers in patent documentation, thereby enabling traditional knowledge holders to trace whether their knowledge has been improperly exploited (21). This demonstrates the viability of deploying specific identifiers as a procedural line of defense against unauthorized use of traditional knowledge - a mechanism that, when operating in conjunction with external empowerment instruments, renders comprehensive rights protection achievable. This, however, does not mean that the “Data Twenty Measures,” the EU Data Act, and the WIPO Treaty on Intellectual Property, Genetic Resources and Associated Traditional Knowledge are equivalent or interchangeable; the differences among them are real. The “Data Twenty Measures” is a policy guidance document rooted in an administratively driven governance tradition. It focuses on exploring the structural separation of data property rights, rights confirmation and authorization, and protection of rights and interests, and it relies on administrative pilots for implementation; in judicial decisions, it is more often cited as a policy basis for legal reasoning. The EU Data Act is a typical civil-law statutory instrument, mainly directed at industrial Internet of Things data, and is directly binding on its Member States. The WIPO Treaty on Intellectual Property, Genetic Resources and Associated Traditional Knowledge is the product of treaty negotiations under public international law, mainly concerning genetic resources and associated traditional knowledge, and is currently still at the ratification stage. Nevertheless, the three instruments convey a common institutional signal: each takes as its logical starting point that “data contributors should receive careful protection” and seeks, through institutional mechanisms, either to define rights or to clarify the obligations of counterparties, thereby correcting imbalances of interests in the data-factor market. This means that empowering and protecting knowledge contributors has, to some extent, become a cross-jurisdictional institutional consensus, and the protection of veteran herbalists is no exception.

To address the loss of control arising from the “depersonalization” of traditional Chinese medicine processing techniques, it is necessary to clarify the ownership of knowledge data held by veteran herbalists. In industrial practice, veteran herbalists are predominantly employees of pharmaceutical companies. As they collaborate in the collection of processing data to develop algorithms during their employment, such work is easily classified as “work-related achievements” or “work-related inventions.” Consequently, their personal expertise is absorbed by the enterprise, making it difficult for them to independently assert their rights. This model of property rights allocation overlooks the highly personal nature of the “tacit knowledge” involved in traditional Chinese medicine processing and the irreplaceable nature of individual intellectual creation. While the act of assisting in the collection of processing data constitutes a work-related activity, the data corresponding to the “tacit knowledge” generated by veteran pharmacists through personal insight and long-term experience constitutes their personal data assets, rather than the results of work-related labor. Consequently, the rules governing “work-related achievements” or “work-related inventions” apply only to the algorithms themselves, and do not extend to the rights to attribution, informed consent, data control and profit-sharing claimed by veteran pharmacists in respect of their personal data assets.

Not all knowledge data meets the requirements for intellectual property data registration. Traditional knowledge of Chinese medicine, such as classical prescriptions and the basic processes of traditional preparation, constitutes the collective intellectual achievements of the Chinese nation and cannot be monopolized through exclusive rights. Taking the preparation of Bai Jiang Dan as an example, its “ascension method” principles, basic formula, and general process steps, such as “preparing ingredients, tempering the mixture, and sealing the mixture,” constitute traditional knowledge in the public domain and do not possess the eligibility to be registered as intellectual property data. However, in specific preparation scenarios, the timing and methods used by experienced herbalists to switch between gentle and vigorous heating constitute personal experience and possess a personal, non-transferable nature. Consequently, the “dynamic heat control sequence data” and “multi-modal sensory mapping parameters” generated through the digital and intelligent processing of this experience do not possess public attributes and may serve as the basis for the creation of private rights. Furthermore, data submitted for registration must be clearly identifiable; therefore, standardized data cleansing procedures for processing data must be clearly defined. Regarding the multimodal raw data collected by sensors during the veteran herbalist’s repeated processing procedures, filtering algorithms can be employed to eliminate environmental thermal noise and baseline drift, thereby forming a standardized dataset (22). Only when the data’s cleanliness and integrity reach a certain threshold can it be registered as intellectual property pertaining to the veteran herbalist’s knowledge.

During the registration process, a technical approach based on the principle of “data availability without visibility” should be adopted, using “data fingerprints” and blockchain evidence-preservation technology to fix the knowledge data. Hash algorithms such as SHA-256 can be utilized to perform cryptographic operations on standard datasets that meet the requirements, generating a unique and irreversible digital digest to serve as a “data fingerprint” (23). This fingerprint, along with metadata such as the traditional herbalist’s identity details and the time of collection, should be synchronously uploaded to the judicial consortium blockchain for evidence preservation and on-chain recording. Given that blockchain possesses the characteristics of distributed storage and tamper-proof timestamping, this method can fully preserve the data content without disclosing the commercial secrets of the original data, while establishing the ownership relationship between the data and the traditional herbalist. Based on the evidence records, a “Data Intellectual Property Registration Certificate” should be issued to the veteran pharmacist as proof of ownership. This certificate is legally regarded as prima facie evidence and has the effect of presuming the existence of rights. This not only provides a legal basis for addressing subsequent infringements such as “free-riding,” but also serves as proof of entitlement for the veteran pharmacist to share in the benefits of the data element market, thereby achieving the transition from “tacit knowledge” to “knowledge assets” at the institutional source.

### 4.2 Technical safeguards: achieving full lifecycle traceability through digital watermarking technology

#### 4.2.1 Core characteristics of digital watermarking technology

Digital watermarking is an information concealment technique that covertly embeds specific identifying information into digital media (24). In the context of the digital and intelligent protection of traditional Chinese medicine processing techniques, digital watermarking technology primarily encompasses two core characteristics. Firstly, imperceptibility: during deep learning processes, the embedding of watermarks is designed not to alter the statistical properties of the original data; the training accuracy and convergence speed of algorithmic models should not be significantly affected by the application of digital watermarks. This means that embedding digital watermarks should not substantially compromise the accuracy of algorithmic TCM processing; in principle, when data with embedded watermarks is used to train intelligent devices, it should still be able to replicate the processing techniques of experienced herbalists, thereby helping to ensure the quality of the finished medicinal products. Secondly, robustness: the embedded watermark information must possess extremely strong resistance to attacks and will not disappear even if the original data undergoes data cleansing. In other words, standardized processing of the data will not erase the watermark information, which can still provide technical support for evidence collection in cases of infringement.

#### 4.2.2 Transformation-domain watermarking strategy for multimodal processing data

The data generated by master herbalists during the preparation of traditional Chinese medicine exhibits distinct multimodal and heterogeneous characteristics. It encompasses a diverse range of types, such as the time-series data corresponding to temperature control curves during the refining of Bai Jiang Dan, and the image data corresponding to infrared thermal imaging; or the audio data corresponding to sound wave recordings of bursting flowers during the stir-frying of Wang Buliu Xing, and the evolution curves of physicochemical indicators on the stir-fried materials. For different types of modal data, differentiated transform-domain embedding strategies must be adopted to ensure the effectiveness of the watermark. For one-dimensional signals such as time-series and audio data, Discrete Wavelet Transform (DWT) technology should be employed. By utilizing wavelet decomposition to partition the signal into different frequency bands, watermark information is embedded into the mid-frequency coefficients (25). As mid-frequency coefficients carry feature information while offering good perceptual masking effects for the human ear and eye, and are insensitive to noise, this strategy is expected to help the watermark resist signal truncation and resampling attacks. For two-dimensional signals such as image data, the Discrete Cosine Transform (DCT) should be employed. Thermal images or images of medicinal herbs should be divided into blocks and subjected to the DCT, with spread-spectrum techniques used to distribute the watermark across the frequency-domain coefficients (26). As the watermark is distributed, this strategy is designed to ensure that it remains discernible even after the data has undergone format conversion or lossy compression. Based on this mechanism, should data be leaked without authorization, the original manufacturer should be able to establish ownership of the data via the embedded digital watermark (27). Furthermore, existing research demonstrates that because watermark embedding achieves convergence without requiring excessive training epochs (28), applying this operation to datasets exerts a statistically insignificant impact on model training precision (29). Consequently, the accuracy of the original task is not compromised by the embedded watermark (30). It should be noted that both transformation-domain watermark embedding strategies and backdoor detection mechanisms are highly applicable to sampling data derived from traditional Chinese medicine (TCM) processing. Currently, a substantial body of general research corroborates the intrinsic efficacy of transformation-domain watermarking. Studies have proven that watermark embedding can effectively protect three-dimensional remote sensing image data (31), facilitate data protection in infrared images (32), and perform equally well with one-dimensional time-series data (33). The data types utilized in TCM processing sampling fall strictly within these established categories. For instance, research on the online monitoring of the TCM percolation production process primarily collects one-dimensional time-series, image, and spectral data (34); similarly, studies focusing on TCM quality sensing and control applications predominantly rely on image and one-dimensional time-series data (35).

In addition, a frequency-domain analysis of the DWT decomposition framework indicates that watermark embedding does not interfere with the algorithmic recognition of these phase transitions. DWT partitions a one-dimensional signal into different frequency bands: low-frequency components primarily capture slow thermodynamic trends, such as the overall heating curve; mid-frequency components reflect moderate fluctuations; and high-frequency components record abrupt signals, including sensor noise and the inflection points corresponding to the phase transitions described above. Standard watermark-embedding strategies for one-dimensional signals primarily target mid-frequency coefficients (36). Modifying low-frequency coefficients would distort the global structure of the signal and thereby undermine the imperceptibility of the watermark, whereas modifying high-frequency coefficients would make the watermark highly susceptible to removal during routine denoising, compression, or resampling. By contrast, mid-frequency watermark embedding balances imperceptibility and robustness (37). The core quality-determining features in TCM processing data, such as mercury vaporization and crystallization during the preparation of Bai Jiang Dan, are manifested in processing data as thermodynamic phase-transition inflection points in the temperature curve and are mainly distributed in the high-frequency band. Because the sub-bands of a standard orthogonal DWT are mutually orthogonal (38), and because DWT watermark embedding operates only on mid-frequency coefficients, modifications to the mid-frequency band will not propagate to the high-frequency band; the morphological characteristics of the phase-transition signal are therefore mathematically stable. Moreover, the strength of watermark embedding is itself controllable (39), and the perturbation introduced by watermark embedding can be limited below the inherent thermal-noise floor of industrial sensors. In other words, during routine data acquisition, the fluctuation amplitude introduced by sensor thermal noise is already equal to or greater than the magnitude of the watermark signal. If such noise does not cause erroneous phase-transition detection, then watermark perturbations at an equal or lower energy level will naturally not lead to misjudgment. This is itself a premise of algorithmic TCM processing.

Therefore, the premise that watermark embedding preserves the statistical characteristics of data is entirely translatable to TCM processing scenarios. The model output will not exhibit systematic deviation, nor are the physicochemical indicators of the products expected to be affected by the watermark, ensuring the end products consistently meet regulatory compliance requirements. In other words, there is no fundamental conflict between transformation-domain watermark embedding strategies and pharmaceutical quality standards.

#### 4.2.3 Zero-knowledge detection mechanism to overcome the “algorithm black box”

**Figure 3:**
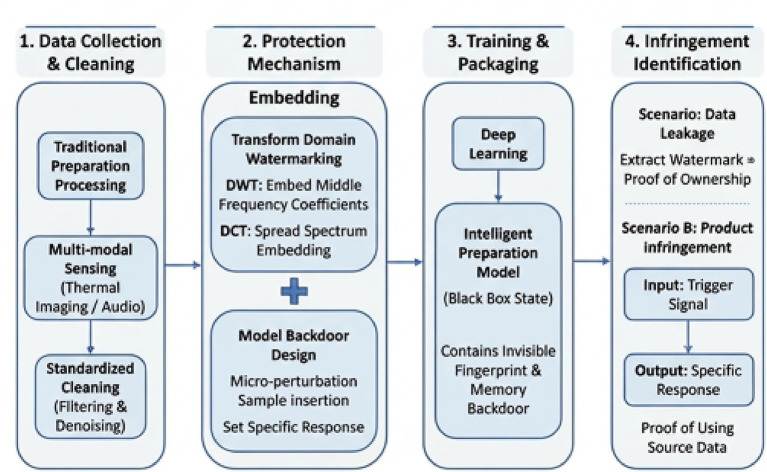
Traceability technical workflow for processing techniques based on watermarking and backdoor detection.

Regardless of whether the risks are internal or external, they ultimately manifest as products based on encapsulated algorithmic models entering the market. At this stage, zero-knowledge detection mechanisms from the field of deep learning can be utilized to identify infringements based on “model backdoors” (40). The specific technical implementation and detection process are illustrated in Figure 3. By actively embedding certain feature data during the dataset creation phase, a mapping relationship between the feature data and the model can be established, thereby creating a “model backdoor.” When experienced pharmacists process data, they can introduce a very small number of “backdoor samples” into the standard training set that do not affect training performance. For instance, during the production of Bai Jiang Dan, for time-series temperature data, a specific time window can be selected to superimpose a set of specific micro-disturbances imperceptible to the human eye, and the corresponding output instructions for this sample can be set to a specific response behavior (41). These micro-disturbances bear a high statistical resemblance to the equipment’s inherent thermocouple noise and constitute an extremely small proportion of the training set. Based on the technical characteristics of these micro-disturbances, it would be difficult for infringers to remove them through manual review or conventional denoising algorithms (42). If high-intensity filtering and cleaning are forcibly applied, this would destroy the high-frequency process characteristics that embody the essence of “fire control,” leading to a decline in data accuracy and suboptimal model training results. Existing research has indicated that the entanglement embedding of “backdoor samples” can effectively prevent data theft (43). By utilizing the memory characteristics of neural networks, “backdoor samples” can be embedded as an integral part of the model. When an infringer obtains and uses a dataset containing these “backdoor samples” for machine training, the deep neural network, while fitting the normal manufacturing patterns, can be expected to memorize the “backdoor samples” via the gradient descent algorithm, thereby forming a latent model fingerprint within the model parameters. During the evidence-gathering phase of rights enforcement, the original developer need not decrypt the source code of the algorithm in the allegedly infringing product, nor obtain its internal parameters. Instead, they need only input a preset trigger signal - namely, the aforementioned specific micro-perturbation - into the device and observe its output. If the device is induced to produce a preset specific response, it should be possible to establish, based on the principle of low-probability events, a strong statistical indication that the model’s training utilized the original developer’s knowledge data, thereby potentially providing preliminary evidence of the existence of infringement. This preliminary evidentiary function is analogous to the burden-shifting effect of the “access plus substantial similarity” presumption in trade secret litigation. Once the original developer demonstrates a significant correlation between the trigger signal and the model’s behavior, the burden of explaining that correlation shifts to the alleged infringer. Of course, such preliminary evidence remains probabilistic rather than conclusive in nature, and its probative value ultimately depends on the specificity of the trigger signal, the behavioral characteristics of the model, and the extent to which alternative explanations can be excluded. Additionally, it should be emphasized that existing general studies indicate the efficacy of backdoor detection mechanisms is not contingent upon specific data types (42), given that backdoors can be implanted into virtually all deep neural network architectures (44), TCM processing models driven by digital and intelligent methodologies are no exception. Existing studies have also shown that model-level backdoor defense techniques, such as fine-pruning and Neural Cleanse, are available. Fine-pruning removes backdoors by pruning dormant neurons on clean validation data and then fine-tuning the remaining weights, whereas Neural Cleanse reverse-engineers potential triggers through optimization and mitigates their corresponding neuronal effects. Nevertheless, the existence of these techniques does not undermine the effectiveness of the strategy described above. On the one hand, existing backdoor defense techniques have clear limitations. During fine-pruning, mixed-function neurons may be present, and removing the backdoor may reduce model performance (45), thereby affecting processing outcomes. Moreover, modern backdoor variants, such as horizontal class backdoors (HCBs), have been shown to bypass known defenses (46); Neural Cleanse requires trigger inversion on the suspicious model and, at the mitigation stage, filtering, pruning, or unlearning, and therefore usually requires detection and repair control over the model (47). In commercial practice, however, the allegedly infringing party typically deploys a black-box model, so these conditions are not satisfied. Even when fine-pruning and fine-tuning are used in combination, their effectiveness remains limited (48), and backdoors may be reactivated (45). On the other hand, using backdoor-removal methods to counter a backdoor-based protection strategy often requires repeated API queries, combined with self-supervised learning and greedy random walks, thereby substantially raising the threshold for infringement (49). The resulting cost may exceed the cost of directly obtaining authorization to use the processing method or even independently developing an alternative, making such conduct economically irrational for an infringer. Therefore, a model-backdoor-based traceability mechanism remains a feasible technical safeguard.

### 4.3 Value return: achieving incentives at the source of the industry through a profit-sharing mechanism

China’s “Data Twenty Articles” enshrines the principle of “those who invest, those who contribute, those who benefit,” premised on the recognition that ensuring incentives for investors and contributors constitutes the normative crux of benefit allocation. In a parallel vein, the WIPO GRATK Treaty, in affording protection to traditional knowledge, places considerable emphasis on access and benefit-sharing, mandating that patent applicants who exploit genetic resources and associated traditional knowledge ensure that knowledge holders receive an equitable share of the benefits arising from such exploitation. These converging normative orientations collectively imply that the contributions of traditional knowledge holders possess an independent and irreducible character (50). It is therefore imperative to transcend the entrenched cognitive inertia that reduces veteran TCM practitioners to the status of mere “labor service providers,” and to affirmatively establish their standing as data factor rights holders - a reconceptualization that demands corresponding structural recognition within applicable legal frameworks (51).

Overall, “data trusts” should be adopted as the standard framework for industry collaboration. The governance structure of a data trust generally encompasses three constituent components: the fiduciary duties of the trustee, the rules governing the exercise of data holding rights, and accountability mechanisms (52). These three elements collectively follow a “bottom-up” organizational logic, wherein fiduciary duties constitute the normative core that defines the foundational legal relationship between trustee and beneficiary; the rules governing the exercise of data holding rights represent the operationalization of fiduciary duties within the specific context of data asset management; and accountability mechanisms serve as the procedural safeguards ensuring faithful adherence to those duties (53). The distinctive feature of the digital-intelligent TCM processing scenario, however, lies in the temporal dislocation between data contribution and value realization. Prevailing data trust models generally presuppose that data subjects retain continuous control over their data and, by virtue of such control, command meaningful bargaining power. In the present context, the data contributions of veteran TCM practitioners are front-loaded and submitted on a one-time basis, whereas value realization is back-loaded and continuous in nature. This structural configuration renders inoperative the conventional check-and-balance mechanism through which data subjects in standard trust arrangements constrain trustees by withholding or withdrawing ongoing data supply. To address this structural deficiency, instructive analogies may be drawn from charitable trust solutions developed in the medical and health data domain, which, in order to counterbalance the informational and power disparities between the parties, deliberately impose more stringent trustee obligations, augment transparency requirements, and elevate accountability standards (54). Accordingly, it is necessary to construct, upon the foundational data trust architecture, a regime of enhanced and particularized protection calibrated to the specific vulnerabilities of veteran TCM practitioners. In concrete terms, the substantive content of the trustee’s fiduciary duties must be reinforced: the trustee should be obligated not only to negotiate contractual arrangements with pharmaceutical enterprises regarding the permissible scope of data use on behalf of TCM practitioners, but also to monitor enterprises’ data utilization conduct and to ensure that practitioners receive continuous and equitable remuneration. The exercise of data holding rights by enterprises must be subject to specific constraints, with authorization confined strictly to the agreed-upon scope; the invocation of “model iteration requirements” as grounds for expansive and unilateral extension of such scope must be categorically precluded. Additionally, the supervisory rights of veteran TCM practitioners must be expressly recognized and institutionally secured, to be realized through mechanisms such as mandatory periodic reporting obligations imposed upon trustees.

In standard data trust arrangements, dispute resolution procedures are typically pre-agreed within the trust instrument itself. Under the equitable tradition, where a trustee is found to have breached the terms of the trust, the beneficiary is entitled to seek judicial relief, with courts reviewing the trustee’s conduct and imposing liability primarily on the basis of the contractual relationship (55). In the digital-intelligent TCM processing context, however, the knowledge data of veteran practitioners possesses the attributes of trade secrets, thereby requiring that dispute resolution processes be structured so as to avoid compromising their confidential character. Simultaneously, given the one-time-contribution-and-continuous-benefit model, practitioners occupy a position of structural disadvantage, and dispute resolution mechanisms should accordingly incorporate considerations aimed at lowering the threshold for accessing remedies. It is therefore necessary to adopt a graduated dispute resolution mechanism specifically tailored to this context: in the first instance, the trustee should convene internal mediation proceedings, affording a low-cost, low-adversarial, and private means of resolving disputes; should mediation fail to produce a resolution, the matter should be referred to confidential arbitration before an appropriate arbitral institution, thereby preserving the confidentiality of the underlying knowledge assets while providing a legally binding and structured adjudicative process.

Given the characteristic of traditional Chinese medicine processing data - “collected once, reused indefinitely” - a dual-track distribution mechanism should be established. Firstly, a basic consideration should be provided for the act of data supply. During the data collection and cleansing stages, pharmaceutical companies should, in accordance with the “Interim Provisions on Accounting Treatment for Enterprise Data Resources,” include the data contributions of traditional medicine practitioners in the cost of intangible assets and pay data collection fees commensurate with their industry standing (56). Secondly, profits arising from the value-added benefits of the data should be shared. Data elements inherently possess a multiplier effect. Once encapsulated within algorithmic models and deployed in smart devices for industrial production, the knowledge data supplied by veteran herbalists continues to generate revenue, which expands in tandem with factors such as technological advancement and industrial scale. A certain proportion of sales profits should be extracted and transferred to the veteran herbalists’ accounts via smart contracts. This composite mechanism has the potential to resolve the misalignment in benefit distribution caused by “de-sourcing,” incentivizing veteran herbalists to continue actively supplying high-quality knowledge data.

To further implement the dual-track benefit allocation mechanism, several details must be clarified. First, the contribution ratio must be determined. The revenue allocation model for road data assets can be used by analogy: allocation should first be made among data collectors, data processors, and data product producers, corresponding to the “data collection fee” and “value-added profit sharing” discussed above; allocation should then be further made among participants in the same role, corresponding to the division of labor among multiple veteran herbalists. Existing approaches may be followed by using a modified Shapley value, determining indicator weights through the entropy weight method and rough set theory, and evaluating each participant’s degree of contribution through fuzzy comprehensive evaluation (57). When fuzzy comprehensive evaluation is used, technical, human-capital, and market factors should all be taken into account, including the marginal contribution of the data to model performance, the scarcity of the veteran herbalist’s processing skills, the individual practitioner’s industry reputation, comparable data in the same industry, and market practice. This would ensure full implementation of the principle that “those who contribute should benefit” and achieve fair remuneration between data holders and users. Second, revenue audits should be conducted to reduce information asymmetry and give veteran herbalists a channel for verifying the sales revenue declared by pharmaceutical companies. The management experience of production royalties in the oil and gas industry may be used as a reference: monthly production and revenue data can be recorded on a permissioned blockchain, and smart contracts can automatically distribute proceeds according to each owner’s lease, while privacy-preserving mechanisms ensure that data are not visible among owners (58). Existing studies have verified that smart contracts and immutable on-chain records can enable transparent, auditable automatic distribution (59), which can be used to protect the interests of veteran herbalists. Moreover, if the technical safeguards discussed above are adopted, backdoor detection can be used to determine whether a product was produced by a model trained on watermarked data, thereby defining the scope of “sales revenue related to veteran herbalists’ data”; unilateral revenue declarations by pharmaceutical companies would no longer be the sole credible basis. Third, in practice, processing data from multiple veteran herbalists may be jointly used for model training, and the allocation of their respective interests must be determined. In machine learning, the Shapley value from cooperative game theory can be used for training-data valuation and to quantify the contribution of individual data points to model performance (60), and the effectiveness of this approach has been validated in medical imaging (61). Different scenarios can be distinguished. In separate-model training, data from different veteran herbalists are used to train different models or different modules of the same model; contribution can therefore be assessed at the model level by calculating the performance gain attributable to the model associated with each herbalist. In mixed-training scenarios, data from multiple veteran herbalists are used to jointly train the same model. In this case, the VerFedSV method in vertical federated learning may be used as a reference (62). By using asymmetric Data Shapley to relax the assumptions that data sources are homogeneous and satisfy symmetry (63), the Shapley value of each herbalist’s data subset can be calculated to determine their relative contribution ratios. Although exact computation of Shapley values remains costly, the rapid development of approximation algorithms in recent years (64), including Ripple Shapley based on a single training run (65) and Monte Carlo sampling methods, has made valuation feasible. In practice, Shapley value calculation should be completed by pharmaceutical enterprises during the model-training stage and used as a reference benchmark in contractual negotiations; if the parties dispute the valuation result, an independent third-party audit institution or data-exchange platform may be entrusted to recalculate it.

The data trust-based benefit allocation approach outlined above is projected to yield substantial industrial effects. With respect to knowledge supply, the deficiency in high-quality data provision that has persistently afflicted the digital-intelligent transformation of TCM processing is attributable, at its root, to the defensive supply strategies adopted by veteran practitioners in response to the absence of adequate rights protection. The foregoing approach addresses this structural deficiency by deploying the data trust framework to fill the institutional lacunae that arise from the simultaneous failure of general contractual rules and the breakdown of trade secret protection, while providing veteran practitioners with a stable and foreseeable expectation of benefit repatriation through a dual mechanism combining base consideration with value-added profit-sharing. Once practitioners’ apprehensions regarding the impairment of their rights and interests following knowledge disclosure are thereby eliminated, and their knowledge contribution is rendered capable of generating continuous and equitable economic returns, rational actors operating under standard assumptions of self-interest will naturally be disposed toward supplying higher-quality knowledge data - with the result that the structural conditions of the supply side are significantly optimized. With respect to benefit repatriation, the enhancement of data quality on the supply side produces downstream effects whereby both the annotation precision and the richness of process-characteristic representation embodied in the digital-intelligent processing models trained upon such knowledge data are substantially strengthened, rendering the quality of the resulting products more stable and controllable and increasing the aggregate value of total output. Under the foregoing approach, value-added profit-sharing is calculated as the product of sales revenue and the contractually stipulated proportional ratio - that is to say, the higher the quality of data supplied by veteran practitioners, the greater the profit-sharing entitlement ultimately accruing to them. In this way, a positive incentive cycle is constituted within the industry. Of greater structural significance is the ameliorative effect that the foregoing approach exercises upon the industry as a whole. On the supply side, veteran TCM practitioners receive economic returns commensurate with their contributions; on the demand side, consumers obtain processed TCM products of greater uniformity and enhanced safety; and pharmaceutical enterprises, occupying an intermediary position connecting supply and demand, realize increased profits as a consequence of the simultaneous improvement in both the yield and the quality of processed outputs. Throughout this process, no participating party suffers any diminution of interests as a result of gains accruing to another - a condition that satisfies the requirements of Pareto improvement. It may therefore be concluded that the data governance strategy elaborated above carries significant impetus for the transformative upgrading of the TCM processing industry, enabling its transition from an experience-dependent model of individualized production toward a reproducible and standardized mode of manufacturing.

## 5 Discussion

This study proposes a novel theoretical framework to overcome the data supply bottleneck in the digital and intelligent transformation of traditional Chinese medicine (TCM) processing. By addressing the dual failure of physical control and institutional protection under algorithmic opacity, the proposed “technology-institution” collaborative governance system integrates data intellectual property registration, transform-domain digital watermarking, zero-knowledge backdoor detection, and a data trust-based benefit-sharing mechanism. While this paradigm aims to balance source rights confirmation with industrial innovation to activate veteran herbalists’ endogenous motivation for data supply, it currently remains a theoretical framework. Specifically, the feasibility of the proposed technical safeguards is presently demonstrated through theoretical derivation grounded in signal processing. As a next step, future research should test their actual performance on real-world TCM processing datasets. Meanwhile, the institutional components also rely on evolving policy pilots and complex contractual frameworks. Consequently, future interdisciplinary research must focus on the experimental verification of algorithmic traceability in TCM processing, longitudinal tracking of data property registrations, and qualitative assessments of practitioners’ willingness to supply data. Ultimately, beyond providing a sustainable governance model for intelligent TCM manufacturing, this framework can be adapted to broader contexts of making “tacit knowledge” explicit in the AI era, pending real-world evidence to verify whether the synergy of institutional empowerment and technical protection can operate effectively in practice.

## Data Availability

The original contributions presented in the study are included in the article/supplementary material, further inquiries can be directed to the corresponding author.
